# Muscle agonist–antagonist interactions in an experimental joint model

**DOI:** 10.1007/s00221-012-3227-0

**Published:** 2012-08-29

**Authors:** Andrei V. Gorkovenko, Stanislaw Sawczyn, Natalia V. Bulgakova, Jaroslaw Jasczur-Nowicki, Viktor S. Mishchenko, Alexander I. Kostyukov

**Affiliations:** 1 Department of Movement Physiology, A.A. Bogomoletz Institute of Physiology, National Academy of Sciences, Bogomoletz Str. 4, Kiev, 01024 Ukraine; 2 University of Physical Education and Sport, Kazimierza Gorskiego str 1, 80-336 Gdansk, Poland

**Keywords:** Muscle antagonists, Hysteresis, Movement after-effects, Motor control

## Abstract

The experiments presented here and performed in anaesthetized cats aimed at studying the dynamics of interactions between antagonist muscle groups. The tendons of *triceps surae* muscles of both hindlimbs were connected with an artificial joint (a pulley installed on a shaft). The muscles were activated by the distributed stimulation of five filaments of cut ventral roots *L*7–*S*1 on both sides of the spinal cord; movements were evoked by the rate-modulation of the stimulation trains. The study mostly compared programs of reciprocal activation and co-activation, including different changes in stimulation rates of muscle antagonists. The most common feature of the movements in both activation modes was hysteresis of the joint angle changes in dependence on stimulus rate. *Reciprocal activation* appeared suitable for a precise regulation of both amplitude and velocity of the movements in direction of the agonist shortening; maximal effectiveness was achieved during full switching off the antagonist stimulation at plateaus of the movement traces. The reverse movements during decrease of the agonist’s stimulation rate demonstrated an explicit nonlinear form with pronounced initial phase of the joint angle fixation. The *co*-*activation* pattern distinctly reduced the hysteresis of joint movements and suppressed the stimulation after-effects, such as the lasting residual movements after fixation of the stimulation rates.

## Introduction

The dynamics of skeletal muscle contraction is essentially nonlinear in that it depends not only on the instantaneous values of neural activation and external load, but also on the direction of previous movement and activation prehistory, demonstrating complex hysteresis-like features (Partridge and Benton 1981; Kostyukov 1987, 1998; Herzog et al. 2006). Contractions of agonist and antagonist muscle groups generate movement around a limb joint, and during any movement, the muscle antagonists change their lengths in opposite directions. Since the dynamic muscle properties crucially depend on the direction of length change, the joint dynamics will reflect complex interactions of the direction-dependent asymmetries in behavior of the muscle antagonists. It seems that the role of muscle hysteresis is traditionally underestimated in motor control investigations; it is considered predominantly in studies devoted to the analysis of cyclic muscle contractions in conditions close to isometry (Weiler and Awiszus 2000; Gillard et al. 2000; Politti et al. 2003; Finni 2006).

The patterns of activation of antagonistic muscle groups may differ not only in different motor tasks, but may significantly vary even in identical movements, depending on a balance between the activation intensities of the antagonists. It is quite clear that for a given movement amplitude, the required level of agonist activation will depend on the intensity of antagonist activation. Indeed, real movements quite often contain elements of co-activation. It is commonly accepted that co-activation of antagonists increases the mechanical stiffness of the joint, what is especially important for complex multi-joint movements (Dounskaia 2005). Increased stiffness is also important for overcoming joint instability under varying external loads; co-activation of antagonistic muscles is one of the main factors improving movement precision (Gribble and Ostry 1998; Gribble et al. 2003).

During active shortening of the agonist, three main patterns in central commands targeting the antagonist may be distinguished. In the first pattern, the increasing activity of agonists is accompanied by a decrease in activity of antagonists, and this pattern is here designated as *reciprocal* *activation*. Secondly, a movement may also be performed by simultaneously changing the activities of agonists and antagonists in the same direction (i.e., rise–rise or drop–drop); this stimulation pattern is here referred to as *co*-*activation*. In addition, it is also necessary to consider movements evoked by active contraction of agonists under *constant activation* of antagonists. In view of the nonlinear muscle dynamics, the three patterns of agonist–antagonist activation can be expected to differ considerably when required to produce the same joint movement.

In simplified form, the muscle dynamics can be considered as a nonlinear system whose output, muscle length, depends on two input variables, intensity of neural activation and external load. The simplest way to analyze such systems is to clamp one of the inputs and record reactions evoked by a step or ramp-and-hold change of the other input (Luenberger 1979). Such an analysis was presented in earlier papers (Kostyukov 1987; Kostyukov and Korchak 1998). Cyclic changes of one input variable evoke pronounced hysteresis-like trajectories of muscle length, and the hysteresis is observed even at very low velocities of the input signal. In addition, the muscle hysteresis has pronounced after-effects in that very different equilibrium lengths are attained after movements in opposite directions (Kostyukov 1987, 1998; Herzog et al. 2006). Increasing the velocity of the input change (external load or stimulation rate) leads to addition of dynamic components to the hysteresis loops, substantially widening them as compared with quasi-static movements (Kostyukov 1987).

The muscle dynamics crucially depends on movement direction. Muscle shortening, evoked by ramp-and-hold unloading of a steadily activated muscle, could be satisfactorily described by an analytical approximation of the movement trajectories within a wide range of the ramp velocities (Kostyukov 1987). The lengthening processes are much more complicated and unpredictable, especially at high amplitudes and velocities of load change. This creates additional difficulties for the evaluation of force–velocity dependencies in eccentric muscle contractions (Rack and Westbury 1974; MacIntosh and Holash 2000).

Quality of theoretical predictions for the real movement trajectories seems to be closely dependent on correctness in choosing the experimental grounds for the modeling. If to take into account only the static (isometric) characteristics of the antagonistic muscles acting around a joint, it would be impossible to predict the trajectories at various patterns of activation. In our opinion, this problem inevitably arises if one uses equilibrium point hypothesis proposed by Anatol Feldman in the 1960s of the last century (Feldman 1966; Hogan 1985; Feldman and Levin 2009). The theory considers the equilibrium positions of a joint with using quasi-static characteristics of the stretch-reflexes for the antagonist muscles. However, it was shown that the muscle hysteresis itself was essentially increased in the stretch-reflex system; thus, instead of similar quasi-static loading characteristics for two antagonist muscles, it would be more preferable to use temporal combinations of the stretch and unloading reflexes for these muscles changing their lengths in opposite directions (Kostyukov 1998). Even if to refuse from accounting the reflexes at preliminary stage of analysis of the single-joint movements, one should possess information on possible mechanical interaction of the hysteresis effects in the muscles. The present study has been undertaken as an initial step in getting such experimental data. In addition, we would like to analyze a hypothesis that some of undesirable consequences of the hysteresis effects in the system of antagonistic muscles could be somewhat diminished due to a mutual compensation.

Therefore, the dependence of muscle dynamics on movement direction complicates the analysis of joint dynamics due to opposite length changes of antagonistic muscles. To simplify such an analysis, we here start with an artificial joint having a simplified geometry for arrangement of the muscles antagonists. This approach had first been applied earlier to define stationary states in agonist–antagonist interactions during various order of their activation (Kostiukov 1986). In the present study, movements around the artificial joint were evoked by more complex patterns of stimulation, with a focus on *reciprocal activation* or *co*-*activation* of antagonistic muscles in the absence of external loads.

## Materials and methods

### Preparation

Experiments were carried out on four adult cats of either sex weighting 2.9–3.5 kg. Animals were purchased from a state-controlled animal farm through the common animal facility of A.A. Bogomoletz Institute of Physiology (Kiev); the use of the animals was approved by the Ethics Committee of the Institute and performed in accordance with the ethical standards laid down in the Helsinki Declaration (1964). Animals were anaesthetized with pentobarbital sodium (initial dose 45 mg/kg i.p. with additional i.v. injections when needed). Catheters were inserted into an external jugular vein (for infusion of necessary fluids and drugs) and a common carotid artery (for monitoring the blood pressure). The *triceps surae* muscles of both hindlimbs were separated from surrounding tissues; their tendons were extracted with small pieces of calcaneus. All the limb nerves except for those to the muscles under study were cut. A laminectomy was performed in the region of the lumbar enlargement of the spinal cord. On both sides, the L_6_–*S*_2_ ventral roots were dissected and cut near the spinal cord. The animal was suspended within a firm frame; the tibia and knee joint were rigidly fixed. The prepared muscles were placed in the bath formed from surrounding skin, wrapped loosely into cotton bandage, and irrigated continuously with heated Ringer solution. A bath filled with mineral oil was made around the exposed spinal cord. Temperatures in both baths were kept close to 37–38 °C by means of radiant heating. The rectal temperature was maintained at a constant physiological level through controlled heating of the animal body with a heating pad. At the end of all experiments, the animals were killed by an overdose of pentobarbital sodium (5 ml of 60 mg/ml solution).

### Recording, data acquisition, and analysis

For simplicity, the prepared triceps surae muscles under study will be referred to as “flexor” (*f*) and “extensor” (*e*). They were connected via Dacron strings with an artificial “joint”, consisting of a pulley that was rigidly mounted on a revolving shaft. The shaft was installed horizontally behind the animal’s hindlimbs and was fixed by bearings placed within two racks. The pulley and shaft were prepared from a lightweight aluminum alloy in order to minimize a moment of inertia of the system. The shaft was coupled with a sensor measuring its rotation, that is, “joint angle”. The pulley diameter was 20 mm, its maximal rotation over 10^0^ thus corresponded to a change in muscle length of 3.5 mm. Amplitudes of the maximal muscle stretches never exceeded 11 mm above the resting length; thus, the evoked movements did not exceed physiological range of the length changes for *m*. *triceps surae* in cats.

The muscles were activated by distributed stimulation of five filaments of cut ventral roots *L*7–*S*1 on both sides of the spinal cord. This method consists in continuous cyclic distribution of higher rate stimulation between the efferent filaments, thus imitating a natural pattern of the efferent activity arrived to the muscle under study (Rack and Westbury 1969). The root filaments were selected so that electrical stimulation of each caused isometric contraction strength between 1.4 and 2.6 N. Amplitudes of the single contractions evoked by stimulation of various filaments were adjusted by equalizing their amplitudes at the level of the minimal value recorded during supramaximal stimulations of the filaments by turns. After the readjustment in intensity of stimulation, the stimulation currents applied to single ventral root filaments remained unchanged throughout experimental procedure. The resulting forces in the muscles were not specially equalized during rate-modulated distributed stimulations, and at the same stimulation rate one of the muscles usually generated larger force than another. For simplicity, a stronger contracting muscle was designated as “flexor” (or “agonist”), while a weaker one was considered as “extensor” (“antagonist”); positive direction in the “joint angle” change corresponded to shortening of “flexor”.

The rate-modulated patterns of stimulation were generated by two DAC channels of the card PCI/PXI-6711 supported by LabVIEW 9 program (National Instrument, USA). Two homemade electronic devices were used for cyclic distribution of the rate-modulated pulses via five channels with regulated output currents. The method of distributed stimulation allowed to achieve fused muscle contractions at relatively low rates of stimuli applied to a single filament (Rack and Westbury 1969, 1974). In the present experiments, no additional external loads were applied to the joint.

**Fig. 1:**
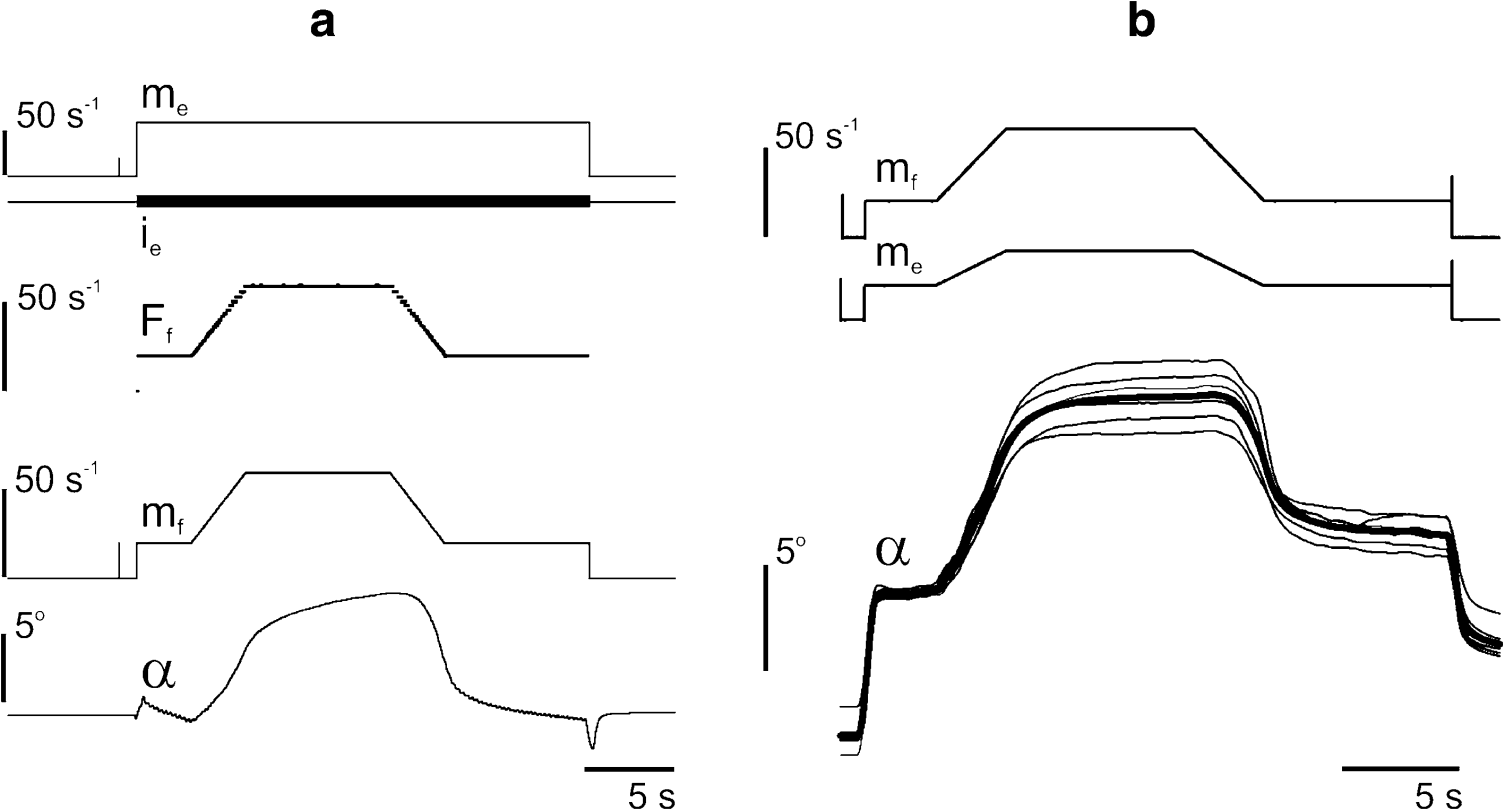
Presentation of the signals recorded in the experiments. **a** The following signals were registered online: the changes of joint angle, *α*; the modulation signals *m*_*f*_, *m*_*e*_ that were used by the developed software to generate pulse trains *i*_*f*_, *i*_*e*_ for stimulation of the “flexor” and “extensor” muscles, respectively. Also controlled were the instantaneous rates of the generated impulses (*F*_*f*_, *F*_*e*_) before they entered the devices of distributed stimulation (see “Materials and methods”). Note that the stimulation rates applied to separate nerve filaments were five times lower than the input rates *F*_*f*_, *F*_*e*_, which, for a sake of simplicity, were presented by the modulation signals *m*_*f*_, *m*_*e*_. **b** A standard set of signals used for further analysis of the movement parameters: the modulation signals *m*_*f*_, *m*_*e*_; α—six repetitions of joint angle traces (*thin lines*) and their average (*thick line*)

Data were collected by CED Power 1401, using program Spike 2 (Cambridge Electronic Design, UK). Origin 8.0 (OriginLab Corporation, USA) and SPSS 17.0 (IBM Business Analytics software) were used for analysis of the experimental data. The following signals were recorded: joint angle (α); modulation signals defining the program of stimulation of the muscles *m*_*f*_, *m*_*e*_; pulse trains (*i*_*f*_, *i*_*e*_) entering the devices of distributed stimulation; instantaneous rates of the pulse trains (*F*_*f*_, *F*_*e*_) (Fig. 1). Figure 1 shows that instantaneous rates of stimulation are quite similar to the modulation signals, and this allowed us to use them further for the sake of simplicity. Standard 2-min intervals of rest were inserted between successive tests.

### Statistical analysis

All records were obtained by sixfold repetition of the same sequences of consecutive tests. The trajectories belonging to identical tests were averaged and stored for following statistical analysis. For example, the lower panel of Fig. 1b shows superimpositions of six movements (thin traces) and their average (thick trace). Impacts of the experimental conditions on the variables were tested by one- and two-way ANOVA. Two-way ANOVA was used for analysis of variables that were compared on phases of rise and decrease in stimulation rate (the definition of such variables is given in legends to Figs. 4, 5, 7). One-way ANOVA was applied for variables, which were not directly associated with specific phases of the rate change, such as the hysteresis loop area.

The stimulation pattern (*S*), direction of change in stimulation rate (*D*), as well as their interaction (*S* × *D*) were compared by two-way ANOVA. Factor *S* had four or five levels depending on the quantity of tests in a given experiment. Factor *D* consisted of two levels (increase or decrease of the stimulation rate). For pair comparisons, the Bonferroni post hoc test was used. Homogeneity of variances was tested by Levene’s test, with a *p* of 0.05 being assumed statistically significant (i.e., *p* < 0.05 of Levene’s test implies a significant inequality of standard deviations). Statistical analysis was performed by SPSS 17.0 (IBM Business Analytics software).

## Results

### General comparison of movements generated by *reciprocal* and *constantactivation* patterns

**Fig. 2:**
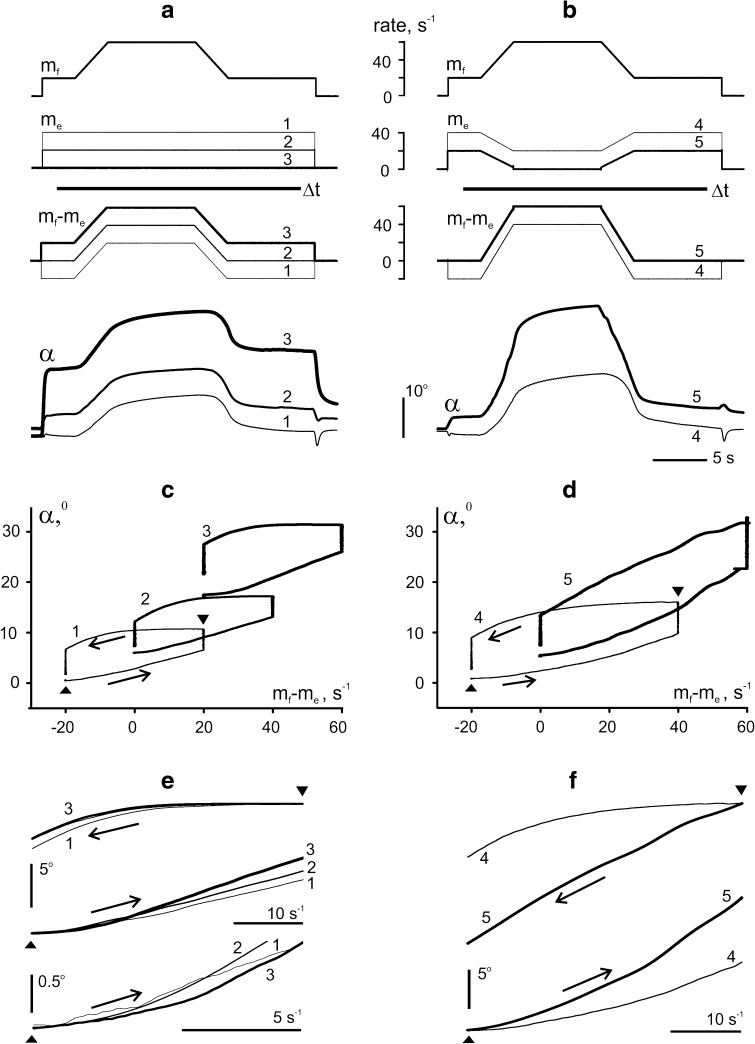
Comparison of the averaged movement traces in response to sequences of five different programs of *e*-stimulation (*m*_*e*_, 1…5), each of which was applied simultaneously with the same program of *f*-stimulation (*m*_*f*_). The sequences were repeated six times, and the records obtained with identical programs of stimulation were averaged off-line. **a**, **b** Modulation signals of *f*- and *e*-stimulations (*m*_*f*_, *m*_*e*_), differences between these signals (*m*_*f*_  − *m*_*e*_), and average movement traces; the *numbers above the records* refer to a definite stimulation program. **c**, **d** Plots of the averaged joint angle, *α*, versus difference of the modulation signals (*m*_*f*_ − *m*_*e*_) (hysteresis loops). *Left column* (**a**, **c**, **e**): tests with two different levels of *constant* *e*-stimulation (1, 2) and without *e*-stimulation (3). *Right column* (**b**, **d**, **f**): two programs with decreasing rates of *e*-stimulation (*reciprocal activation*). *Lines* Δ*t* in *panels* **a**, **b** indicate the sampling interval used for drawing the hysteresis loops (*panels* **c**, **d)**. **e**, **f** Superposition of the direct (ascending) and reverse (descending) branches of the hysteresis loops in tests 1–3 (**e**) and 5–6 (**f**), such that their initial points coalesced as marked by up- and down-directed triangles, respectively. Additionally, the initial parts of the combined direct branches 1–3 are presented in a larger scale at the *bottom* *of panel* **e**

Figure 2 demonstrates results from an experiment designed to compare *reciprocal* and *constant* *activation* patterns. The programs of *f*-stimulation were identical in all tests, duration of the tests consisted of 25 s. The modulation signal *m*_*f*_ included an initial and final steady rate of 20 s^−1^, superimposed on which was a trapezoidal (ramp-and-hold) rate increment. The trapezoid had symmetric edges lasting 3 s, and the rate at its apex consisted of 60 s^−1^. Tests 1–3 (left column) represent examples of *constant* *activation* patterns, including various levels of a steady *e*-stimulation (Fig. 2a, c, e). Tests 4–5 (right column) represent examples of *reciprocal activation* patterns, with *m*_*e*_ decreasing in a trapezoidal manner, which mirrored the trapezoids of *m*_*f*_ (Fig. 2b, d, f). The rate differences between *f*- and *e*-stimulations are presented as *m*_*f*_ *−* *m*_*e*_.

The movement record (*α*) obtained for a passive antagonist (*m*_*e*_ = 0) is shown in Fig. 2 a as the thick trace labeled 3. Its main features correspond to the reactions of an isotonically loaded muscle under activation patterns similar to *f*-stimulation in this experiment (Kostyukov and Korchak 1998). A slow development of movement at the initial stage of the rate increase was followed by acceleration, and the subsequent movement then became almost linear. During the rate fixation on the plateau of the trapezoid in *m*_*f*_, the movement continued, gradually slowing down, until the end of this plateau. During the succeeding rate decrease, the angle was firstly unchanged, then movement in backward direction slowly accelerated and its trajectory became almost linear. At the final rate of 20 s^−1^, the movement relaxed near-exponentially to an equilibrium value. There was a clear mismatch between the angle values at the beginning and final phases with the same stimulation rates. In the angle-rate plot, *α*_*3*_(*m*_*f*_ *−* *m*_*e*_) in Fig. 2c, the mismatch is expressed as a rupture of the hysteresis loop at the left vertical side (trace 3 in Fig. 2c). The difference between the initial and final values of the joint angle depended on the level of antagonist activation, diminishing from 4.18 ± 1.11 (test 3, thick lines) to 1.41 ± 1.21 (test 2, medium-thick lines) and 1.23 ± 0.92 (test 1, thin lines), the decreases being statistically significant in both cases (*p* < 0.05). During constant activation of antagonists at rates of 20 and 40 s^−1^ (traces 2 and 1, respectively, in Fig. 2a, c), the averaged trajectories shifted to lower angle values, their ranges shrunk, and the corresponding hysteresis loops became narrower. The initial parts of the loops (bottom curves in Fig. 2e) suggest that the beginning points of movement acceleration were shifted to the left in transition from passive state of antagonist (3rd test) to its activation (2nd and 1st tests). Thus, in movements generated by *constant* *e*-*stimulation*, an increase in the activation rate of the antagonist muscle appeared able to shorten the delays of the following movement along the direct branches of the hysteresis loops, having almost linear form. At the same time, the reverse movements during rate decrease remained essentially nonlinear, showing a long-lasting fixation of joint angle at their beginning.

Opposite changes in activation levels of agonist and antagonist muscles occur quite often in real motor tasks. This situation was modeled as shown in the right column of Fig. 2. While the temporal profile of agonist stimulation rate (*m*_*f*_; Fig. 2b, upper panel) was the same as in the left column, the stimulation patterns of the antagonist (*m*_*e*_; Fig. 2b, middle panel) were mirror images of the agonist pattern. This combination will be referred to as *reciprocal activation*. As compared to *constant* *e*-*stimulation* (Fig. 2, left column, traces 1–3), *reciprocal activation* pattern (Fig. 2, right column, traces 4, 5) increased the movement amplitudes, as to be expected. This is most evident in test 5 (thick lines), in which the antagonist rate (*m*_*e*_) fell to zero during the plateau phase of the trapezoidal rate change. During *reciprocal activation*, the reverse branches of the hysteresis loops showed a lesser extent of deflection from linearity (compare Fig. 2d with Fig. 2c). When the antagonist rate (*m*_*e*_) fell to zero (thick line), the reverse movement began very quickly after resumption of antagonist stimulation and had almost linear form. In addition, despite the larger amplitude of movement, the difference between the initial and final equilibrium joint angles was smaller than in the test with a passive antagonist muscle (compare size of the gaps at the left vertical parts of hysteresis loops presented by thick traces 5 and 3 in Fig. 2c, d).

### Quantitative analysis of movements evoked by *reciprocal activation* patterns

**Fig. 3:**
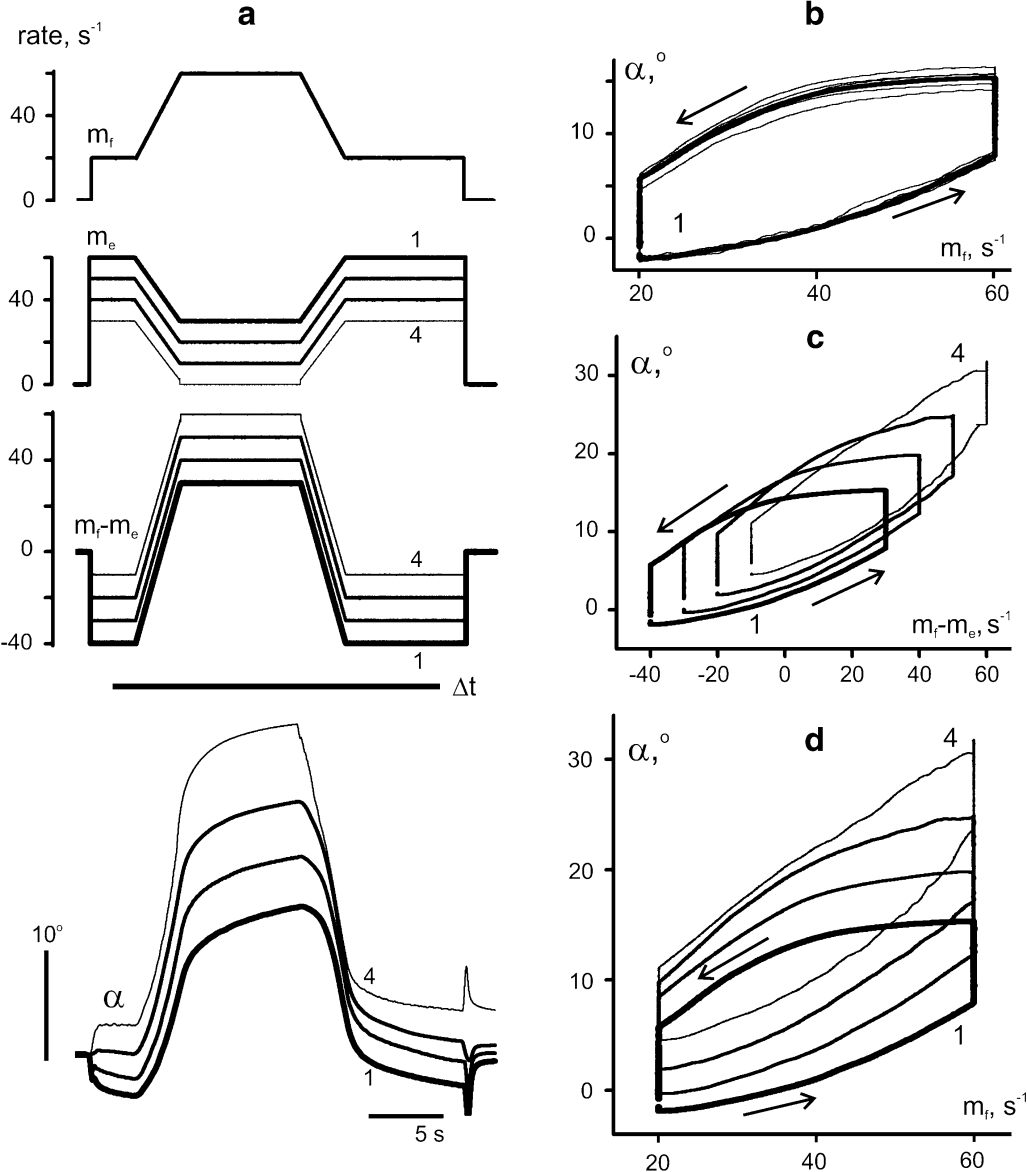
Movement trajectories elicited by four different programs of *e*-stimulation and the same program of *f*-stimulation. **a** Modulation signals *m*_*f*_, *m*_*e*_, their difference *m*_*f*_ − *m*_*e*_, and the averaged records of joint angle, *α*. **b** Dependence of the averaged joint angle on *f*-stimulation rate (*thick line*) and the underlying single records (*thin lines*) that were used for statistical analysis of the movement parameters. **c**, **d** Hysteresis loops *α*(*m*_*f*_ –*m*_*e*_) and *α*(*m*_*f*_)

**Fig. 4:**
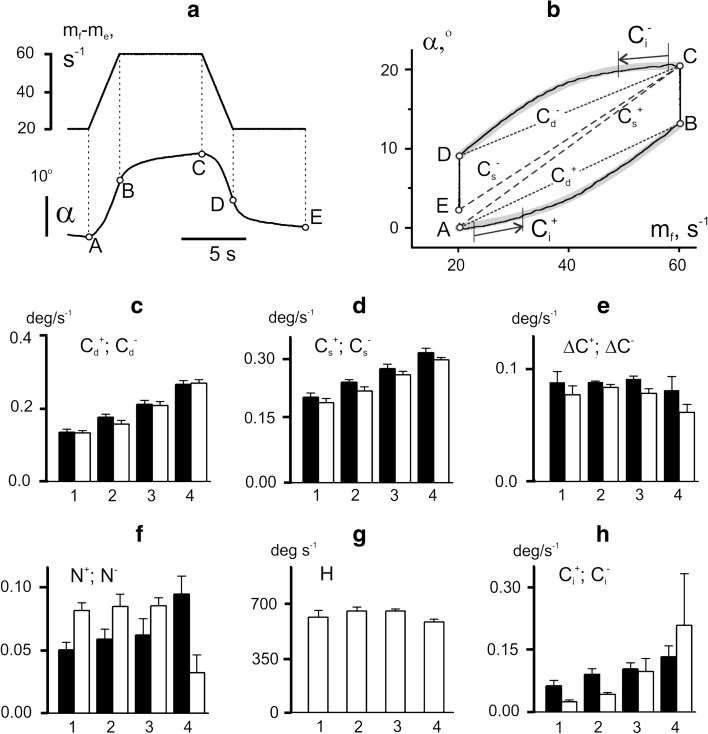
Statistical characteristics of the evoked movements from the experiment shown in Fig. 3. **a**, **b** Explanation of the analyzed parameters with using the averaged movement trajectories recorded in the 1st test of the experiment in Fig. 3. **c**–**h** Statistical characteristics (m ± SD) of the following parameters are presented in the plots: *C*_*d*_^+^, *C*_*d*_^−^—indexes of dynamic compliance (**c**); *C*_*s*_^+^, *C*_*s*_^−^—indexes of static compliance (**d**); Δ*C* ^+^ = *C*_*s*_^+^ − *C*_*d*_^+^, Δ*C* ^−^ = *C*_*s*_^−^ − *C*_*d*_^−^—differences between the above indexes (**e**); *N* ^+^, *N* ^−^—amplitudes of nonlinear distortion of the direct and reverse branches in the hysteresis loops (**f**); *H*—areas of the hysteresis loops (**g**); *C*_*i*_^+^, *C*_*i*_^−^—slopes of the initial part of hysteresis branches (**h**). The parameters relating to the direct and reverse branches of the hysteresis loops are shown by *black* and *white bars*, respectively (**c**–**f**, **h**). A more detailed description of the parameters is given in the text

Figure 3 presents another experiment, in which the same trapezoidal pattern of *f*-stimulation was combined with four patterns of opposite changes of *e*-stimulation (trapezoidal decreases in rate), while their initial and final rates varied. In the fourth test (thin line labeled 4), *e*-stimulation rate fell to zero at its minimal level. To get a more precise quantitative grip on movement changes under various stimulation patterns, a statistical analysis was performed of selected parameters of six individual movement traces obtained with each test. The definition and determination of these parameters are explained in panels *a* and *b* of Fig. 4. Panel *a* illustrates the definition of five points on an individual movement trajectory, which correspond to the respective points on the related hysteresis loop *α*_*i*_(*m*_*f*_) in panel *b*. Pairs of these points are connected by four lines labeled by indexed parameters “C”, which represent the line slopes, that is, the angle changes per unit change in stimulation rate. The upper indexes signify whether they are associated with an increase (+) or decrease (−) in the rate of *f*-stimulation. In analogy with the mechanical term of the “compliance” as reverse quantity to the “stiffness”, we introduced the “rate compliance” defining the angle changes per unit change in activation rate. Examples of such a consideration are given elsewhere (Kostyukov and Korchak 1998; Kostyukov 1998). Additionally, a distinction was made between dynamic and static indexes of the rate compliance. The lower indexes, *d* or *s*, distinguish dynamic or static parameters. The dynamic parameters (*C*_*d*_^+^; *C*_*d*_^−^) relate to joint angle changes occurring only during the changes in rate, while the static parameters (*C*_*s*_^+^; *C*_*s*_^−^) relate to movements that include residual movements after the rate fixation. Thus, the dynamic indexes of rate compliance, *C*_*d*_^+^, *C*_*d*_^−^, were defined as the slopes of the lines connecting the initial and final points of the loops at the leading and falling edges of the stimulation rate, AB and CD. The static indexes of rate compliance, *C*_*s*_^+^, *C*_*s*_^−^, were defined as the slopes of the lines AC and CE (points C, E correspond the angle positions after cessation of residual movements at phases of rate fixation). Finally, the following differences between the indexes of compliance were defined: Δ*C* ^+^ = *C*_*s*_^+^ − *C*_*d*_^+^; Δ*C* ^−^ = *C*_*s*_^−^ − *C*_*d*_^−^. These parameters are associated with the amplitudes of residual movements following the corresponding phases of the rate change.

In addition, we also estimated coefficients of nonlinear deflection (*N* ^+^, *N* ^−^) in respective branches of the rate-angle loops. The coefficients were determined by a nonlinear (sinusoidal) approximation of traces *α*(*m*_*f*_) at rising (↑) and falling (↓) edges of the rate changes:

1 $$ \alpha_{ \uparrow } = \alpha_{A} + C_{d}^{ + } (m - m_{1} ) - N^{ + } (m_{2} - m_{1} )\sin \frac{{(m - m_{1} )\pi }}{{(m_{2} - m_{1} )}};\quad m_{1} \le m \le m_{2} ; $$

2 $$ \alpha_{ \downarrow } = \alpha_{D} + C_{d}^{ - } (m - m_{1} ) + N^{ - } (m_{2} - m_{1} )\sin \frac{{(m - m_{1} )\pi }}{{(m_{2} - m_{1} )}};\quad m_{1} \le m \le m_{2} , $$

where *m*_*1*_ and *m*_*2*_ are the minimal and maximal values of *f*-stimulation rate.

Finally, three further variables were determined from the hysteresis loops: (1) the slopes of the initial parts of the leading and falling loop branches (*C*_*i*_^+^, *C*_*i*_^−^) as measures of initial rate compliance, and (2) the loop area (*H*) as a measure of the hysteresis extent.

All the parameters were determined for each of six realizations of each test, and their means and standard variations were calculated for the following statistical analysis. Various tests were compared by using two-way ANOVA, the factors being the stimulation pattern (*S*), direction of changes in rate of *f*-stimulation (*D*), as well as their interaction (*S* × *D*) (Table 1). The stimulation pattern *S* is considered as a combination of *m*_*f*_ and *m*_*e*_. Pattern of *m*_*f*_ changes was identical in all tests of the given experiment; *m*_*e*_ traces (1–4) had the same profile, differing by the basic levels of rate (Fig. 3a). Factor *D* is considered for the phases of increase (↑) and decrease (↓) of the *f*-stimulation rate.

**Table 1:** ANOVA analysis of the experiment presented in Figs. 3 and 4

Parameters	Factors						Leven’s test	
	(*S*: 1…5)		(*D*: ↑, ↓)		*S* × *D*			
	*F*	*p*	*F*	*p*	*F*	*p*	*F*	*p*
*C*_*d*_	403.624	0.000	3.692	0.062	2.603	0.065	0.478	0.844
*C*_*s*_	289.139	0.000	40.379	0.000	0.352	0.788	0.391	0.902
Δ*C*	11.297	0.000	36.254	0.000	2.521	0.072	2.965	0.013
*N*	2.616	0.064	2.257	0.141	56.622	0.000	1.668	0.145
C_*i*_	16.462	0.000	0.103	0.750	4.220	0.011	11.411	0.000
***H***	**9.491**	**0.000**					**1.209**	**0.332**

Two-way ANOVA was performed for parameters depending on movement direction: *C*_*d*_, *C*_*s*_, Δ*C*, *N*, *C*_*i*_ (see explanations in the text). Two factors, the activation pattern (*S*: 1…4) and the direction of change in rate of *f*-stimulation (*D*: ↑, ↓), as well as their interaction (*S* × *D*), were considered. In addition, the table also includes the Levene’s test of homogeneity of variances for every parameter. The parameter *H* (area of hysteresis loop), depending only on the activation pattern factor, was analyzed by one-way ANOVA (highlighted by bold font)

The experiment in Fig. 3 demonstrates rather complex rearrangements of the hysteresis loops when the background level of *e*-activation was lowered. Successive decreases in the background rate of *e*-stimulation from the first to fourth tests (1–4 in Fig. 3a) led to increases in movement amplitude and augmented the indexes of dynamic and static compliances at both branches of the rate-angle loops (Fig. 4c, d). Two-way ANOVA showed that factor *S* influenced both parameters, while factor *D* affected only the index of static compliance (Table 1). Both *S* and *D* factors influenced the difference between the indexes of static and dynamic compliance (∆*C* ^±^), and this parameter, associated with the reverse phase of movement, decreased noticeably during transition to the last test. Therefore, *reciprocal activation* patterns may diminish the after-effects of the reverse phases of the evoked movements, especially when the antagonists are inactive at their apexes.

The nonlinear distortions of the hysteresis loops differed for increases and decreases in the rate of *f*-stimulation. The nonlinear effects were greater in the reverse movement phase in the first three tests. During rate increases, the nonlinear effects increased with decreasing background rate of *e*-stimulation, particularly for the 3rd and 4th tests. During rate decreases, the nonlinear components remained relatively steady in the tests 1–3, decreasing noticeably in transition to the 4th test. If small irregular oscillations at the initial stage of the reverse movement in test 4 are neglected, this section of movement can be considered as almost linear. Similar movement reactions occurred in other experiments (compare test 5 in Fig. 2 and test 4 in Fig. 3). Two-way ANOVA revealed a statistically significant influence of interaction of *S* and *D* factors on the nonlinear effects (Table 1).

The areas of the hysteresis loops (*H*) in this experiment depended on the activation pattern (Table 1), decreasing particularly in the 4th test. In the first two tests (1, 2) with relatively high background rates of *e*-stimulation, the initial rate compliances were more than twice as big for the direct branches of the loops than for the reverse branches. In the 3rd test, the difference vanished, while in the 4th test, an opposite tendency had been appeared.

Ratio between initial compliances *C*_*i*_^+^ and *C*_*i*_^−^ was not constant across various stimulation patterns; at the same time, both parameters demonstrated evident tendency of growth with decrease in the basic rate of *e*-stimulation (Fig. 4h). These parameters showed a significant non-homogeneity of variances according to Levene’s test (*F* = 11.411, *p* < 0.001, see Table 1).

### Movements evoked by *reciprocal activation* patterns with identical plateau rates of *f*- and *e*-stimulations

**Fig. 5:**
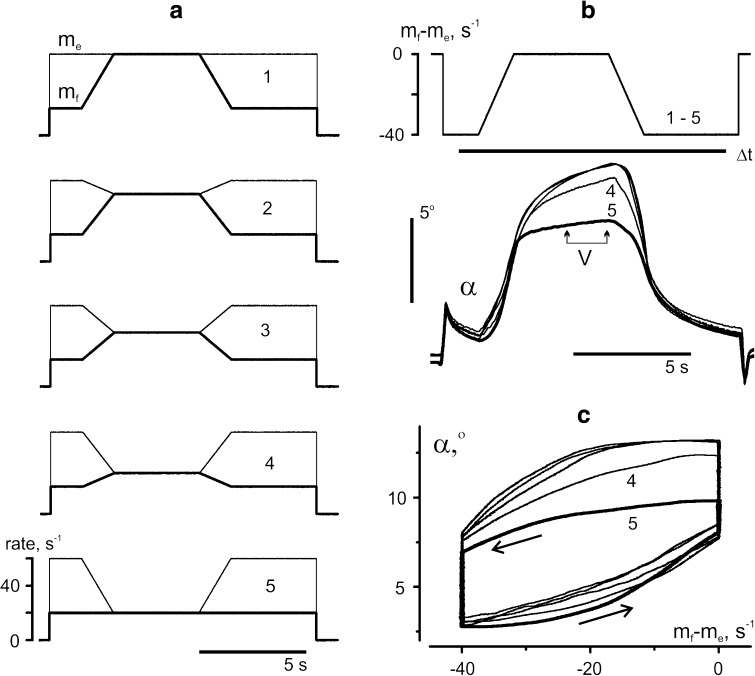
Movement trajectories evoked by five different programs of stimulation. **a** *f*- and *e*-stimulation rates (*thick* and *thin lines*); the rates were changed in opposite directions in accordance with similar modulation signals of trapezoidal form, coinciding at the top of the trapezoids in all tests. **b** Differences between *f*- and *e*-modulation signals; note the same form for all tests. Other descriptions coincide with those presented in Fig. 3

The experiment presented in Fig. 5 compared movements generated by *reciprocal activation* patterns with identical plateau rates of *f*- and *e*-stimulations. In this experiment, the efficiency of *f*-stimulation (*m*_*f*_, thick lines) was markedly greater than that of *e*-stimulation (*m*_*e*_, thin lines); so even though the *e*-stimulation rate was higher at the beginning of all tests, the resulting movements were not evolving in extension direction. Thus, a well-expressed flexion movement occurred even in the 5th test, when the rate of *f*-stimulation was invariable, such that the movement occurred exclusively because of the drop in *e*-stimulation rate. Interdependent changes in the rates of *f*- and e-stimulations evoked quite similar movements in tests 1–3, in which the trajectories superimposed neatly (Fig. 5b). Moreover, the traces in all tests (1–5) were close to each other during rate increases; then, the movement trajectories of the 4th and 5th tests slowed down during rate fixation, thus diminishing the amplitudes of residual movement.

**Fig. 6:**
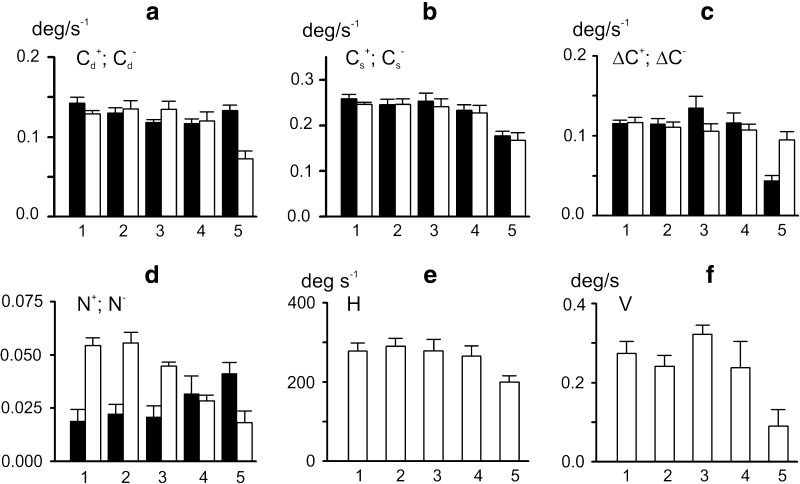
Statistical characteristics of the evoked movements from the experiment in Fig. 5. **a–e** The definition of the parameters was mainly the same as in Fig. 4, a difference being the use of the hysteresis loops *α*(*m*_*f*_ − *m*_*e*_) instead of *α*(*m*_*f*_). **f** The movement velocities that were defined in different tests at the time interval *V* noted in Fig. 5b

The quantitative analysis of the results presented in Fig. 5 is summarized in Fig. 6 and Table 2. Two-way ANOVA revealed a statistically significant influence of the *S* factor on the index of static compliance (Table 2). This parameter showed much similarity in the changes between the direct and reverse phases of movement (Fig. 6b), which is also supported by statistically significant influence of *D* factor on it (Table 2). The index of static rate compliance tended to decrease from the 3rd to the 5th tests. The index of dynamic rate compliance showed a downward trend only for the reversed branch of hysteresis loop (Fig. 6a); two-way ANOVA demonstrated a statistically significant influence of *S*, *D* factors, as well as of their interaction, *S* × *D*, on this parameter. The area of the hysteresis loops decreased significantly only in 5th test, and ANOVA analysis showed that this parameter depended on the *S* factor (Fig. 6e; Table 2). Such a dependence largely corresponds to the above-described behavior of the indexes of static rate compliance. The difference between the indexes of static and dynamic rate compliance decreased significantly for the direct movement branch in the 5th test, what seemed to be directly associated with the reduction in amplitude of the residual movement (Fig. 5b).

**Table 2:** ANOVA analysis of the experiment presented in Figs. 5 and 6

Parameters	Factors						Leven’s test	
	(*S*: 1…5)		(*D*: ↑, ↓)		*S* × *D*			
	*F*	*p*	*F*	*p*	*F*	*p*	*F*	*p*
*C*_*d*_	31.636	0.000	21.404	0.000	42.372	0.000	1.745	0.103
*C*_*s*_	72.395	0.000	5.222	0.027	0.529	0.715	1.309	0.256
Δ*C*	67.008	0.000	0.610	0.438	34.009	0.000	1.866	0.079
*N*	7.910	0.000	104.936	0.000	77.916	0.000	1.330	0.246
***H***	**4,173.867**	**0.000**					**0.504**	**0.733**
***V***	**26.968**	**0.000**					**1.431**	**0.253**

Descriptions as in Table 1. Note that the parameters were defined using loops α(*m*_*f*_ − *m*_*e*_) (see legend to Fig. 5). The sampling interval for velocity calculation (*V*) is presented in Fig. 5b

The nonlinear effects and trends of their change in consecutive tests differed substantially between the direct and reverse branches of the hysteresis loops, increasing in the first case and decreasing in the second (Fig. 6d). Two-way ANOVA revealed that the nonlinear effects depended on the *S* and *D* factors and on their interaction, *S* × *D* (Table 2). The reduction of amplitude of the residual movements at the direct branches in the 4th and 5th tests was correlated with a decrease in their velocity (Fig. 6f). The pattern of stimulation had a statistically significant influence on this parameter (Table 2).

### Movements evoked by *co*-*activation* of the antagonistic muscles

**Fig. 7:**
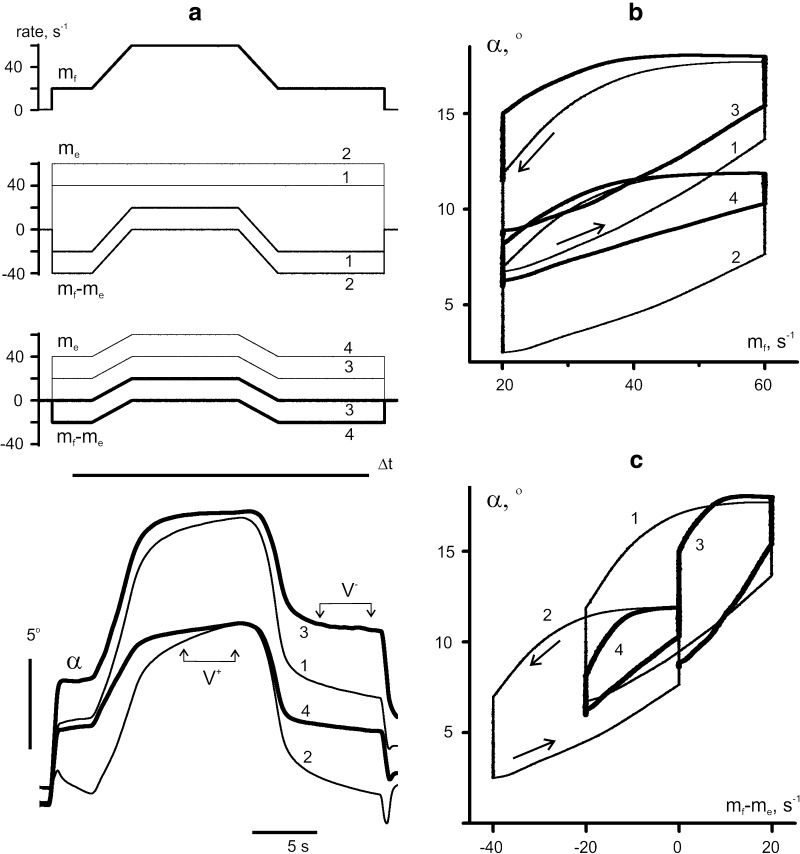
Comparison of averaged movement traces recorded in response to four different programs of *e*-stimulation associated with the same program of *f*-stimulation. **a** Modulation signals *m*_*f*_, *m*_*e*_, their difference *m*_*f*_ –*m*_*e*_, and the averaged records of joint angle *α*. Two constant levels of *e*-stimulation were applied in tests 1 and 2; in tests 3 and 4, *e*-stimulation changed similarly to the program of *f*-stimulation but had two different levels of background rate. **b**, **c** Hysteresis loops *α*(*m*_*f*_) and *α*(*m*_*f*_  − *m*_*e*_)

Many real motor tasks require to *co*-*activate* antagonists simultaneously, such that their activities change in the same direction. An example of movements evoked by *co*-*activation* patterns is shown in Fig. 7. In all tests of this experiment, the same *f*-stimulation pattern was applied (Fig. 7a, upper panel). The evoked movements were compared for *constant e*-stimulations (tests 1, 2) and *co*-*activation* patterns (tests 3, 4) (Fig. 7a, second and third panels). The background rates of *e*-stimulation in the 3rd and 4th tests were chosen in such a way that their maximal rates during trapezoid plateaus coincided with the respective rates in tests 1 and 2. The initial angle values in tests 1 and 2 were lower than those in tests 3 and 4 (Fig. 7a, bottom panel). During the following movements, the respective trajectories (1 and 3; 2 and 4) gradually approached each other; the joint angles in the corresponding pairs of traces almost coincided at their apexes and then split up again, approaching different joint angles before cessation of stimulation.

**Fig. 8:**
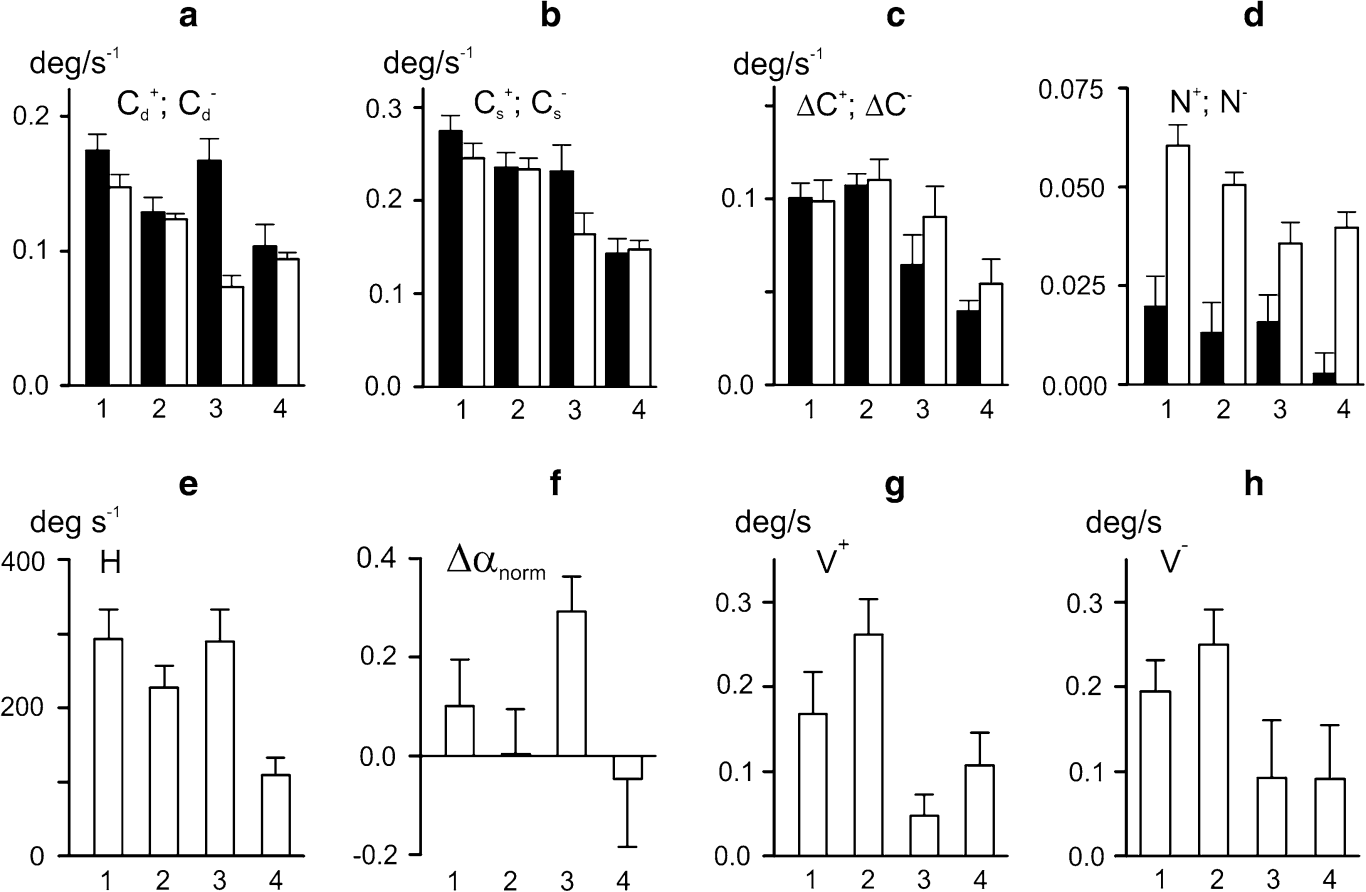
Statistical characteristics of the evoked movements from the experiment in Fig. 7. **a**–**e** Parameters those descriptions coincide with the given ones in **c**–**h** of Fig. 4. **f** The normalized difference between the initial and final values of joint angle at the time interval Δ*t* (normalization with respect to the movement amplitude). **g**, **h** The movement velocities at the time intervals *V* ^+^ and *V* ^−^ noted in Fig. 7a

The quantitative analysis of movements from the above experiment is presented in Fig. 8 and Table 3. Main parameters of the evoked movements were highly dependent on the stimulation pattern (Table 3). Comparison of test 1 (constant *e*-stimulation) with test 3 (*co*-*activation* pattern) did not demonstrate statistically significant differences between the areas of the hysteresis loops (Fig. 8e), while their shapes differed substantially (Fig. 7b). The transition from the 1st to the 3rd test was accompanied by an upward shift of the direct branches of the hysteresis loops without noticeable changes in their shapes. On the other hand, the amplitudes of the residual movements decreased in the 3rd test, so that the averaged traces in the 1st and 3rd tests almost coincided at the beginning of the reverse movement (Fig. 7a). In the 3rd test, the hysteresis loop shrunk (Fig. 7b), and, as it could be seen from Fig. 8c, this accompanied with a statistically significant decrease of the difference between the indexes of static and dynamic rate compliance. In the 3rd test, the hysteresis loop shrunk during the plateau of *f*-stimulation (Fig. 7b), and as seen from Fig. 8c, this was accompanied with a statistically significant decrease of the difference between the indexes of static and dynamic rate compliance.

**Table 3:** ANOVA analysis of the experiment presented in Figs. 7 and 8

Parameters	Factors						Leven’s test	
	(*S*: 1…5)		(*D*: ↑, ↓)		*S* × *D*			
	*F*	*p*	*F*	*p*	*F*	*p*	*F*	*p*
*C*_*d*_	63.942	0.000	110.346	0.000	40.256	0.000	2.303	0.045
*C*_*s*_	91.856	0.000	20.402	0.000	10.180	0.000	1.756	0.124
Δ*C*	64.692	0.000	9.487	0.004	3.220	0.033	1.814	0.111
*N*	23.152	0.000	408.533	0.000	7.867	0.000	1.115	0.373
***H***	**37.191**	**0.000**					**0.955**	**0.433**
**Δα**_**norm**_	**13.063**	**0.000**					**1.523**	**0.239**
***V*** ^**+**^	**31.580**	**0.000**					**0.432**	**0.732**
***V*** ^**−**^	**16.214**	**0.000**					**1.820**	**0.176**

Description as in Table 1. The definition of parameters Δα_norm_, *V* ^+^, *V* ^–^ is given in the legend to Fig. 8

In a similar pair of tests, the 2nd and 4th, the increase in background rate of *e*-stimulation led to a greater difference in the hysteresis effects (Figs. 7b, 8e); the loop area diminished noticeably in the last case. The differences between the indexes of static and dynamic rate compliances were much lower in the 4th than in the 2nd test (Fig. 8c), which reflected a much faster angle fixation in the 4th test (Fig. 7a). The analysis of the nonlinear components in movement traces indicated a high degree of linearity of the direct loop branches recorded in both *constant* and co-*activation* patterns (Fig. 8d). At the same time, the nonlinear components in the reverse loop branches were rather significant. Interestingly, the transition from *constant* to *co*-*activation* patterns reduced the nonlinear distortions in the reverse branches.

The hysteresis causes uncertainty in joint movements at stages of their fixing. The uncertainty can be quantified as normalized difference between the steady values of the joint angles at initial and final rates of *f*-stimulation (normalization with respect to the movement amplitude in the cycle of the rate changes). This parameter was lower for the *co*-*activation* mode (tests 2 and 4) than with *constant* *e*-stimulation (tests 1 and 3). The reduction of this parameter in the *co*-*activation* pattern was also associated with another positive effect, a significant drop in the speed of residual movements at the phases of the rate fixation (Fig. 8h). Two-way ANOVA analysis for the given series of tests showed that *S* and *D* factors as well as their interaction, *S* × *D*, strongly influenced all the parameters under study (Table 3).

### Relative change of movement parameters in different patterns of stimulation

**Fig. 9:**
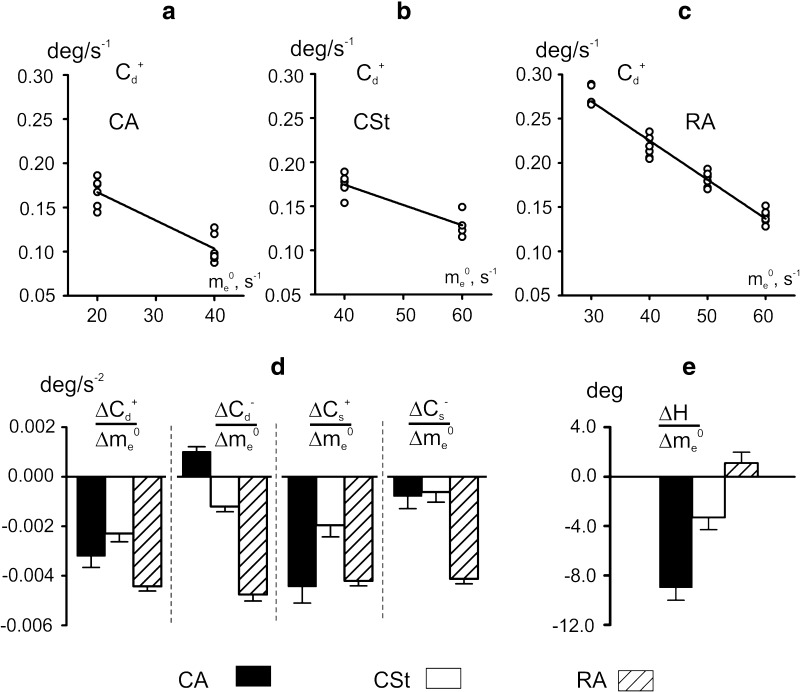
Change of the movement parameters with increase in basic level of the extensor stimulation rate for co-activation (CA), constant stimulation of antagonist (CSt), and reciprocal activation (RA) regimens. The corresponding results are taken from the data presented in Fig. 7 (CA, CSt) and Fig. 3 (RA). **a**–**c** Examples of the linear regression analysis for *C*_*d*_^+^ parameter. **d**, **e** Slopes of the linear regression lines for dependencies of *C*_*d*_^+^; *C*_*d*_^−^; *C*_*s*_^+^; *C*_*s*_^−^; *H* on *m*_*e*_^0^ for the three of stimulation patterns. With excluding CA and CSt comparison in the last set of *bars* (Δ*C*_s_^−^/Δ*m*_*e*_^0^) in **d**, there was observed a statistically significant dependence of the line slopes on stimulation patterns *p* < 0.005 (2 way ANCOVA method)

In Figs. 2, 3, and 7 were considered several sets of movements related to similar *e*-modulation signals that had been differed only by the basic level of the stimulation rate. Increase of *m*_*e*_^0^ mainly diminished amplitudes of the evoked movements, although relative changes of the studied parameters seemed to be different in various stimulation modes. Dependences of the movement parameters *C*_*d*_^+^; *C*_*d*_^−^; *C*_*s*_^+^; *C*_*s*_^−^; *H* on basic level of the extensor stimulation rate *m*_*e*_^0^ were compared for different activation patterns (*co*-*activation*; *constant* *e*-stimulation, and *reciprocal activation*) (Fig. 9). Two-way ANCOVA test was applied to calculate slopes of the regression lines for the three simulation patterns (Fig. 9a, b, c). The activation pattern was taken as one factor, while the basic level of the extensor stimulation rate had been considered as covariate one. The test revealed that all parameters were highly dependent upon pattern of stimulation (*p* < 0.005), decreasing predominantly with rise of *m*_*e*_^0^. The test also revealed that slope of regression line was significantly dependent on pattern of stimulation (*p* < 0.005). If to compare correspondent changes of the dynamic and static indexes of rate compliance at direct phases of movement (relating to rise of *f*-stimulation rate), it could be noticed that the slopes of the regression lines were not essentially differed for *co*-*activation* and *reciprocal activation* regimens ($\frac{{\Updelta C_{d}^{ + } }}{{\Updelta m_{e}^{0} }}$ and $\frac{{\Updelta C_{s}^{ + } }}{{\Updelta m_{e}^{0} }}$ bars in Fig. 9d). At the reverse phases of movement, such a difference had been evidently appeared regimens ($\frac{{\Updelta C_{d}^{ - } }}{{\Updelta m_{e}^{0} }}$ and $\frac{{\Updelta C_{s}^{ - } }}{{\Updelta m_{e}^{0} }}$ bars in Fig. 9d). In this case, noticeably smaller changes of the compliance indexes were recorded in the *co*-*activation* regimen as compared with the *reciprocal* one. *Co*-*activation* regimen occurred to be also more effective in diminishing the hysteresis effects with rise in basic level of the extensor stimulation rate *m*_*e*_^0^ (Fig. 9e).

**Fig. 10:**
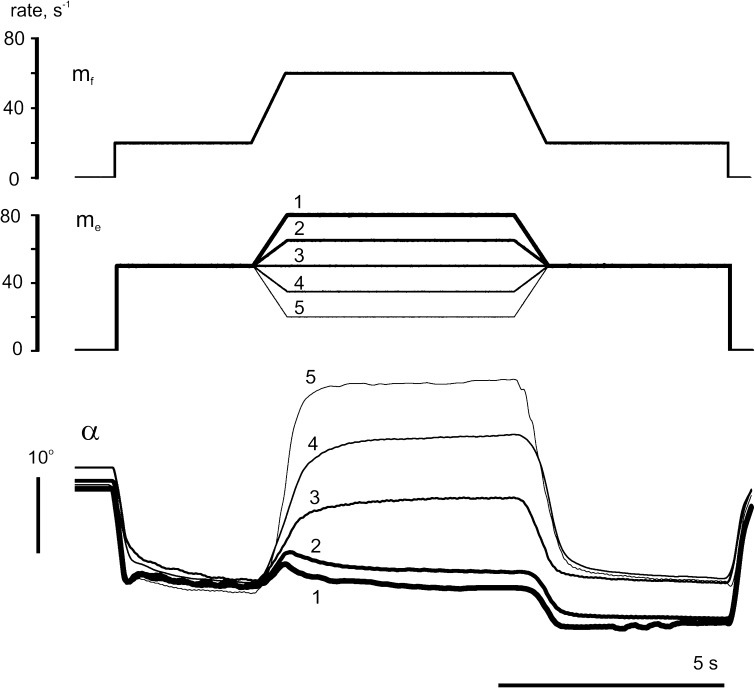
Comparison of three stimulation patterns: *co*-*activation* (traces *1*, *2* of *m*_*e*_ records), *constant stimulation* of antagonist (3), and *reciprocal activation* (*4*, *5*) in an experiment with prevailing activation of the antagonist muscle (movement in extending direction at the beginning stage of *f*- and *e*-stimulations)

The both *co*-*activation* and *reciprocal activation* regimens were considered in the present study from the point of view of their *assistance* to a basic movement in “flexing” direction. For sake of simplicity, the muscle generating more intense contractions was arbitrarily considered as “flexor”. Illustration of another example with predominance of “extension” contraction force is given in Fig. 10. The basic rate of the antagonist stimulation was sufficient in this case to evoke initial movement in “extending” direction; application of different rate-modulated changes of *e*-stimulation allowed to compare main activation patterns: *co*-*activation* (traces 1, 2), *constant* *e*-stimulation (3), and *reciprocal activation* (4, 5). During increases in rate of *e*-stimulation (traces 4, 5), antagonist muscle could easily compensate for the force addition in agonist. Therefore, instead of assisting role of the *co*-*activation* regimen for the case of prevalence in contraction action of the agonist, it would be preferable to note its compensating character when forces generating by the antagonist muscles are close to each other.

## Discussion

### Variability in activation patterns of the muscle antagonists

In the present study, an attempt to analyze a single-joint movement has been undertaken by using various programs of activation of the muscle antagonists evoking movements around an artificial joint. The stimulation rate of “flexor” muscles changed in all experiments according to a trapezoidal modulation signal, whereas three patterns of stimulation rate of “extensor” muscles were compared. The stimulation rate of the “extensor” diminished in *reciprocal activation* pattern and rose in *co*-*activation* pattern, the time course of the rate changes being identical in both cases. In some experiments, the *reciprocal activation* and *co*-*activation* patterns were juxtaposed with *constant* *stimulation* of antagonist.

The most common feature of the movements evoked by all kinds of stimulation was hysteresis. In the absence of antagonist stimulation, the joint movements evoked by trapezoidally varying stimulation rates of the agonist closely resembled the corresponding reactions of isotonically loaded muscles (Kostyukov and Korchak 1998). The initial rise in stimulation rate of the agonist evoked a slow movement followed by a faster, almost linear movement during further rate rise. Other important features of the joint movements were the lasting residual changes in joint angle at the plateau of *f*-stimulation rate and its powerful clamping during the initial stage of the following rate decrease. This finding is consistent with earlier ones showing that if the active shortening is followed by a decrease in stimulation rate, the muscle length remains almost invariable even up to 30–40 % drop of rate (Kostyukov and Korchak 1998). It is interesting that, as compared with passive antagonist, the movements were faster in the initial stages of *f*-stimulation rate increase in any of the used stimulation modes, *constant e*-*stimulation*, *reciprocal activation*, and *co*-*activation* (Figs. 2, 3, 7). An active antagonist thus seems to be crucially important for accelerating initial stage of the joint movement.

### *Reciprocal activation* of muscle antagonists

*Reciprocal activation* of antagonists seems to present a most convenient way to regulate both the amplitude and velocity of the single-joint movements (Figs. 2, 3, 5). When the amplitude of the rate changes remained the same, a decrease in background rate of antagonist stimulation (at the initial and final stages of the tests) could both increase the movement amplitude and achieve a faster movement in opposite direction. Moreover, the reverse movement could exhibit a trajectory linearization, beginning almost simultaneously with the start of reverse change in *f*-stimulation rate (Figs. 3, 4). The *reciprocal activation* mode is likely most effective when the *e*-stimulation is completely switching off at apex of the agonist shortening (compare test 5 in Fig. 2d and test 4 in Fig. 3d). Maximal movement amplitudes are accompanied in this case with diminish of nonlinearity of both direct and reverse phases of movement.

Another important feature of the *reciprocal activation* pattern is probably associated with its flexibility in the execution of various motor tasks. Combinations of substantially different stimulation programs can evoke identical movement trajectories. Particularly effective in this respect appear to be opposite changes of the antagonist activation rates such that their sum remained unaltered (Fig. 5). In a wide range of such changes, the movement traces almost coincided, suggesting that drops in antagonist stimulation rate can effectively substitute for increments in agonist muscle activity. Such a redistribution of activity between antagonists can likely provide an effective mechanism for reducing the undesirable residual movements accompanying active contractions of the agonist muscle. It is known that in order to prevent a long and slow residual muscle movement in isotony, its activation rate must be noticeably reduced after achievement of the desired length (Kostyukov and Korchak 1998, Kostyukov 1998). Such muscle dynamics seem to be a main reason for the generation by the nervous system of powerful dynamic components in efferent activity, which can readily explain the presence of huge phasic EMG components accompanying any sufficiently fast joint movements (Tal’nov et al. 1997, 1999). The *reciprocal activation* patterns can likely diminish intensity of efferent activity to the agonist muscles during sufficiently fast movements. As can be seen in Fig. 5, under certain combinations of antagonist stimulation rates, the amplitudes of residual movements can be substantially reduced, thus accelerating movements in reverse direction.

### *Co*-*activation* of muscle antagonists

*Co*-*activation* of muscle antagonists is widely used in a variety of motor tasks (Minetti 1994; Galloway and Koshland 2002). This pattern can appreciably increase the joint stiffness that is of paramount importance for complex limb movements, when precise positioning of distal segments requires additional fixation of more proximal joints (Laczkó et al. 2006; Zakotnik et al. 2006; Fukashiro et al. 2006). The present study demonstrates that *co*-*activation* patterns can distinctly reduce the hysteresis of joint movements and suppress after-effects, such as the lasting residual movements after the rate fixation (Figs. 7, 8). Basing on conceptions elaborated by Feldman in the framework of the equilibrium point hypothesis (Feldman 1966; Feldman and Levin 2009), the present data can be additionally treated in terms of an uncertainty in installing equilibrium positions in a joint. One can assume that the *co*-*activation* modes can somewhat diminish these undesirable effects. At least partly, this could be explained by the present data demonstrating that the movement-dependent discrepancy between steady states was lower for the *co*-*activation* mode as compared with *constant e*-stimulation (Fig. 8f). The reduction of uncertainty was associated with another positive effect, a significant drop in the speed of residual movement during the rate fixation.

In the real movements, *reciprocal activation* patterns could be at least partly corresponded with reciprocity existing at spinal and supraspinal levels for the neuronal subsystems controlling the flexor and extensor muscles (Jankowska and Lindstrom 1972; Hultborn et al. 1976; Jankowska et al. 2005). On the other hand, a possibility for direct cortical activation of the extensor muscles independently of the flexors (Kostyukov and Tal’nov 1991; Iles and Pisini 1992) can provide independent descending control of the antagonistic muscle groups. Under voluntary control, various combinations of flexor and extensor activities appear possible at least for the cases of non-ballistic movements.

We would like to stress that only slow changes in efferent commands were considered in the present study, and movements were analyzed in the absence of external loads. These issues may be studied by extrapolating the present experimental model. However, it is quite clear that the proposed approach cannot be applied to the analysis of more complex organizational features of the real muscle activities, such as various recruitment patterns of single motor units.

## Conclusions

The most common feature of the movements in both the *reciprocal activation* and *co*-*activation* patterns was hysteresis of the joint angle changes in dependence on stimulation rate. *Reciprocal activation* of antagonists appears especially suitable for the precise regulation of both the amplitude and velocity of joint movements. Under a *reciprocal activation* pattern with absence of antagonist stimulation at apex of the agonist activity, the movements in reverse direction demonstrate fast beginning and linear time course. *Co*-*activation* patterns can distinctly reduce the hysteresis after-effects, such as the ongoing residual movements after clamping of stimulation rate, and thereby reduce the uncertainty, that is, difference between the steady values of the joint angle at initial and final stimulation rates.
